# LncRNA LMCD1-AS1 Interacts with PHF8 to Promote Hepatocellular Carcinoma Resistance to Multikinase Inhibitors

**DOI:** 10.7150/ijbs.129651

**Published:** 2026-03-30

**Authors:** Xiao Yang, Songze Song, Tianxing Ye, Xiujuan Zhang, Ziying Yang, Yue Mi, Yanjie Shen, Yanan Zhang, Jie Liu, Qinong Ye

**Affiliations:** 1 National Key Laboratory of Advanced Biotechnology, Academy of Military Medical Sciences, Beijing 100071, China.; 2 Department of Cell Biology, Key Laboratory of Cell Biology of National Health Commission of the PRC, China Medical University, Shenyang 110122, China.; 3 Key laboratory of Carcinogenesis and Translational Research (Ministry of Education/Beiing), Gastrointestinal Cancer Center, Peking University Cancer Hospital &Institute, Beijing 100142, China.; 4 Key Laboratory of Xinjiang Phytomedicine Resources and Utilization, Ministry of Education, School of Pharmacy, Shihezi University, Shihezi 832002, China.; 5 School of Basic Medical Sciences, Tsinghua University, Beijing 100084, China.

**Keywords:** lncRNA, hepatocellular carcinoma, multikinase inhibitor, drug resistance, epigenetic reprogramming

## Abstract

Resistance to first-line multikinase inhibitors (MKIs) sorafenib and lenvatinib critically limits hepatocellular carcinoma (HCC) treatment efficacy. It remains largely unknown how long non-coding RNAs (lncRNAs) affect resistance to MKIs. Through integrated analysis of resistant HCC models, we identified lncRNA LMCD1-AS1 as a critical driver of MKI resistance. LMCD1-AS1 overexpression correlates with advanced tumor stage, shortened survival, and resistance to MKI therapy in HCC patients. LMCD1-AS1 confers dual resistance to sorafenib and lenvatinib by suppressing apoptosis, while its knockdown restored drug sensitivity. Mechanistically, LMCD1-AS1 directly bind histone demethylase PHF8, promoting H4K20me1 to epigenetically activate oncogenes (e.g., c-Myc, β-catenin) and upregulate lactate dehydrogenase A (LDHA). This triggers lactate overproduction and alters the NAD^+^/NADH ratio, establishing a protumorigenic metabolic state. Crucially, PHF8 ablation reverses LMCD1-AS1-driven resistance, and *in vivo* xenografts confirm attenuated sorafenib efficacy with LMCD1-AS1 overexpression. Our work unveils the LMCD1-AS1/PHF8/H4K20me1 axis as a unified epigenetic-metabolic mechanism underlying MKI resistance, representing a promising therapeutic target and prognostic biomarker for HCC.

## Introduction

The global incidence of hepatocellular carcinoma (HCC) is rising steadily at an annual rate of 1-2%, with projections estimating 950,000-1,050,000 new cases by 2025 1,2. Current first-line therapies for HCC, including multikinase inhibitors (MKIs) such as sorafenib and lenvatinib, remain cornerstone treatments 3-5. However, acquired resistance to these agents poses a critical barrier to effective disease management. This resistance not only compromises therapeutic efficacy but also correlates with poor prognosis, significantly reducing median survival post-resistance and necessitating frequent treatment modifications. These challenges compound treatment costs and increase the risk of adverse effects. Consequently, elucidating the molecular mechanisms underlying sorafenib and lenvatinib resistance is an urgent priority in HCC research.

Long non-coding RNAs (lncRNAs), defined as transcripts >200 nucleotides lacking protein-coding potential, are pivotal regulators of eukaryotic gene expression 6. Studies demonstrate their critical involvement in epigenetic modulation, chromatin remodeling, genomic imprinting, and transcriptional/post-transcriptional control 7-10. In HCC, lncRNAs orchestrate chemoresistance through multifaceted mechanisms, including dysregulated drug metabolism, activation of pro-survival signaling, epigenetic reprogramming, and tumor microenvironment remodeling 11-13. These processes collectively enhance tumor cell adaptability and reduce therapeutic efficacy, positioning lncRNAs as promising targets to overcome drug resistance in HCC 14-16. LIM and cysteine-rich domains protein 1 antisense RNA 1 (LMCD1-AS1) is a recently identified lncRNA 17. Emerging evidence indicates that LMCD1-AS1 promotes cancer cell proliferation, migration, and invasion 18-22; however, its potential role in drug resistance remains unexplored.

Through integrated analysis of resistant HCC models, we identify long non-coding RNA LMCD1-AS1 as a critical mediator of MKI resistance. Clinically, its overexpression correlates with advanced tumor stage and poor survival in HCC patients. Functionally, LMCD1-AS1 confers dual resistance to sorafenib and lenvatinib by suppressing drug-induced apoptosis. Mechanistically, it directly binds histone demethylase PHF8 (plant homeodomain finger protein 8)—a context-dependent epigenetic regulator in cancer 23,24—to erase H4K20 monomethylation (H4K20me1), thereby activating oncogenes (e.g., c-Myc, β-catenin) and reprogramming lactate metabolism through upregulation of lactate dehydrogenase A (LDHA), a key enzyme driving the Warburg effect (aerobic glycolysis) 25-28. Crucially, PHF8 ablation reverses LMCD1-AS1-driven resistance, validating this axis as a therapeutic target.

## Materials and Methods

### Cell lines, vectors and reagents

Human hepatocellular carcinoma HepG2 and MHCC-97H cells were purchased from American Type Culture Collection (ATCC) and tested for mycoplasma contamination. Cells were cultured in DMEM medium (Invitrogen) containing 25 mM glucose and 10% fetal bovine serum (Hyclone) at 37 °C with 5% CO2. The LMCD1-AS1 fragments were generated by PCR amplification. The eukaryotic expression vectors were constructed by inserting PCR-amplified fragments into pcDNA3.0 (Invitrogen). Lentiviral vectors for gene overexpression were obtained by inserting PCR-amplified LMCD1-AS1 sequences into pCDH (System Biosciences, USA). Anti-PHF8, anti-c-Myc, anti-Wnt1, anti-β-catenin, anti-FOXA2, anti-SNAI1, anti-LDHA, and anti-G6PD antibodies were purchased from Proteintech. Anti-β-actin was purchased from Santa Cruz Biotechnology. Anti-FLAG was obtained from Sigma-Aldrich.

### Data collection and analysis

The intersecting differentially expressed lncRNAs (DELs) in GSE140202 dataset and GSE186191 dataset were identified as multikinase inhibitor resistance-related lncRNAs. To generate sorafenib-resistant HCC cells, HUH7 cells were seeded in 6-cm dishes (1 × 10⁵ cells per dish) and initially treated with 1.5 µM sorafenib for 24 h. Surviving adherent cells were then cultured in the presence of progressively increasing sorafenib concentrations (1.5, 3.0, 4.5, 6.0, 7.5, and 9.0 µM), with each concentration maintained for 15-21 days. Following approximately 5 months of gradual drug selection, a stable sorafenib-resistant cell line (hereafter referred to as Sor-R cells) was established.

DELs were analyzed by limma R package, and DEL settings were as follows: False discovery rate (FDR) < 0.05 and |log2 fold change (FC)| > 1. Univariable Cox regression analysis was used to identify the relationship between lncRNAs and overall survival (OS) of 366 HCC patients from The Cancer Genome Atlas (TCGA, http://cancergenome.nih.gov) database (TCGA-LIHC). The survival R package was used to conduct survival analyses based on lncRNA expression and OS or disease-free survival (DFS) of TCGA-LIHC patients within a 5-year follow-up time.

### Cell transfection

For plasmid transfection, 10 μg of target plasmid was diluted in DMEM, mixed with the Lipofectamine 3000 reagent, and incubated for 15 minutes at room temperature before being added to the cells. For siRNA transfection, the siRNA duplex was complexed with Lipofectamine RNAiMAX (10 μL) in DMEM and incubated for 20 minutes at room temperature. Cells transfected with plasmid and siRNA were harvested 24 and 48 hours post-transfection, respectively, for subsequent analysis. The cDNA target sequences of small interfering RNAs (siRNAs) for LMCD1-AS1 and PHF8 are listed in Table S1.

### Total RNA extraction and reverse-transcription quantitative polymerase chain reaction (RT-qPCR)

Total RNA was isolated from PBS-washed cell pellets using TRIzol Reagent (Invitrogen) according to the manufacturer's instructions. Briefly, the homogenate was incubated and mixed with 200 μL chloroform, followed by centrifuging at 12,000 × g for 15 min at 4 °C. The aqueous phase was transferred to a new tube, and mixed with 400 μL isopropanol at -80 °C for 30 min. The RNA pellet was obtained by centrifugation, washed with 75% ethanol and absolute ethanol, air-dried, and finally resuspended in 40 μL DEPC-treated water. The cDNA was synthesized based on total RNA using the 5× Evo M-MLV RT Master Mix (Accurate Biology). qPCR was performed using SYBR Premix Ex Taq Master Mix (Takara). The relative expression of the target gene was calculated based on the CT value, and β-actin was selected as the internal reference protein control. Primer sequences for qPCR were listed in Table S2.

### Cell viability assay

Cells were plated at 5 × 10^3^ cells per well in a 96-well plate, and treated with the indicated drugs. Then 10 µL of CCK-8 reagent (TargetMol) was added to each well and incubated at 37 °C for 1 h. Absorbance was measured at 450 nm using a microplate reader.

### Colony formation assays

HepG2 and MHCC-97H cells were plated at a density of 3,000 cells per well in 6-well plates and cultured for two weeks at 37 °C in a 5% CO_2_ atmosphere. Subsequently, the cells were washed once with PBS, fixed with 4% paraformaldehyde for 30 min, and stained with 0.1% crystal violet for 10 min. The number of colonies (defined as clusters of > 50 cells) was manually counted under a microscope.

### Apoptosis analysis

Apoptosis was performed according to the manufacturer's instructions (CellorLab). A 100 µL aliquot of cell suspension (~5×10^5^ cells) was aspirated. Annexin V-FITC (5 µL) and propidium iodide (PI, 5-10 µL) were added, mixed gently, and incubated at room temperature for 15 min protected from light. Subsequently, 800 µL of binding buffer was added to each tube. Cells were centrifuged gently (300 × g, 5 min), and the supernatant was discarded. The pellet was resuspended in 100 µL fresh binding buffer. Samples were analyzed immediately using flow cytometry.

### Western blot analysis

Cells were centrifuged at 3,000 rpm for 5 min at 4 °C and collected on ice. The cell pellet was lysed in RIPA buffer (BioMed) and mixed with SDS sample buffer (1:1 v/v to RIPA buffer). The mixture was denatured by boiling at 100°C for 15 min, followed by centrifugation at 12,000 rpm for 2 min. The supernatant was loaded onto SDS-PAGE gels for electrophoresis. Following electrophoresis, proteins were transferred to nitrocellulose membranes using semi-dry transfer (16V constant voltage). Membranes were blocked with 5% skimmed milk for 1 h at room temperature, then incubated with primary antibody for 1-1.5 h. After three 5-min washes with TBST, membranes were incubated with HRP-conjugated secondary antibody for 1 h. Following another three 5-min TBST washes, protein bands were visualized using enhanced chemiluminescence (ECL) reagent (Vazyme).

### RNA pull-down assay

Biotinylated LMCD1-AS1 RNA was transcribed* in vitro* using the T7 High Yield RNA Transcription Kit (Vazyme) with Biotin-16-UTP and LMCD1-AS1 DNA template. The reaction was incubated at 37 °C for 2 h, followed by DNase I treatment (37 °C, 15 min) to remove template DNA. Subsequently, 1 × 10^7^ cells were harvested and lysed in ice-cold lysis buffer supplemented with protease/RNase inhibitors. Cell lysates or recombinant proteins were incubated with biotinylated RNA at 4 °C for 2-4 h to form RNA-protein complexes. Streptavidin magnetic beads (Sangon Biotech) were added and incubated for 1 h at 4 °C with rotation. After five washes with lysis buffer, bound proteins were eluted in 1× SDS loading buffer at 95° C for 10 min. RNA-interacting proteins were identified by Western blot or liquid chromatography-tandem mass spectrometry LC-MS/MS (Fitgene).

### RNA immunoprecipitation (RIP)

RIP assays were performed using a commercial kit (Millipore). Briefly, 1 × 10^7^ cells were collected, washed with ice-cold PBS, and lysed in RIP buffer supplemented with protease/RNase inhibitors. A 10% aliquot of lysate was reserved as Input control. The remaining lysate was incubated overnight at 4 °C with magnetic beads pre-coated with a specific antibody. After incubation, bead complexes were washed five times with lysis buffer. RNA-protein complexes were eluted with kit-provided elution buffer, and bound RNA was extracted using TRIzol reagent (Invitrogen). Precipitated RNA was quantified by RT-qPCR.

### Lactate and NAD^+^/NADH Measurement

A total of 1 × 10^5^ cells were seeded per well in a 12-well plate. To measure lactate secretion, the medium was replaced with DMEM without FBS. After incubation for 1 h, the supernatant was harvested, and mixed with the reaction buffer (Biovision). Absorbance was measured at 450 nm using a microplate reader. Lactate levels were normalized to cell counts. For NAD^+^/NADH activity, cells were processed similarly. The supernatant was incubated with NAD^+^/NADH reaction buffer (Biovision) at 37 °C for 30 min. Absorbance was measured at 450 nm and normalized to cell number to determine relative NAD/NADH activity.

### Co-immunoprecipitation (Co-IP)

For transfection-based Co-IP, transfected cells were harvested and lysed using 500 ml of lysis buffer (50 mM Tris pH 8.0, 500 mM NaCl, 0.5% Nonidet P-40, 1 mM dithiothreitol, and protease inhibitor). The lysate was subjected to immunoprecipitation overnight at 4 °C using anti-FLAG agarose beads (Sigma-aldrich). Following three washes with the lysis buffer, bound complexes were eluted with SDS sample buffer. These eluted immunocomplexes were then resolved by SDS-PAGE and subsequently analyzed via Western blot using the specified antibodies.

For examining native protein interactions, cells were lysed directly in 500 ml of lysis buffer. The lysate was immunoprecipitated with the antibody of interest or control serum. After rigorous washing steps with lysis buffer, the captured immunoprecipitates were separated by SDS-PAGE and subjected to Western blot analysis.

### Animal experiments

All animal procedures were conducted in accordance with the Guidelines for the Ethical Use of Laboratory Animals approved by the Animal Ethics Committee of the Academy of Military Medical Sciences. HepG2 cells stably expressing LMCD1-AS1 or empty vector were subcutaneously injected into 6-week-old male BALB/c nude mice. Seven days later, intraperitoneal injections of sorafenib (40 mg/kg) or an equal volume of saline control were administered once weekly for 3 weeks. Tumor volume was measured every 3 days, and tumor growth curves were generated. On day 27, all mice were euthanized by carbon dioxide inhalation. Tumors were excised, photographed, and weighed.

### Statistical analysis

Statistical analyses of all experiments were performed using SPSS 13.0 and GraphPad Prism 9.0 software, and all experimental results were repeated three times to obtain similar results. Comparisons between two groups were analyzed using the two-tailed Student's *t*-test. For comparisons among multiple groups, one-way analysis of variance (ANOVA) was applied, followed by Tukey's post hoc test. The normality of data distribution was confirmed using the Shapiro-Wilk test, and homogeneity of variances was verified using Levene's test. Data were expressed as mean ± standard deviation and were considered statistically significant when *P* < 0.05.

## Results

### LMCD1-AS1 is identified as a MKI resistance-related lncRNA

**Figure 1:**
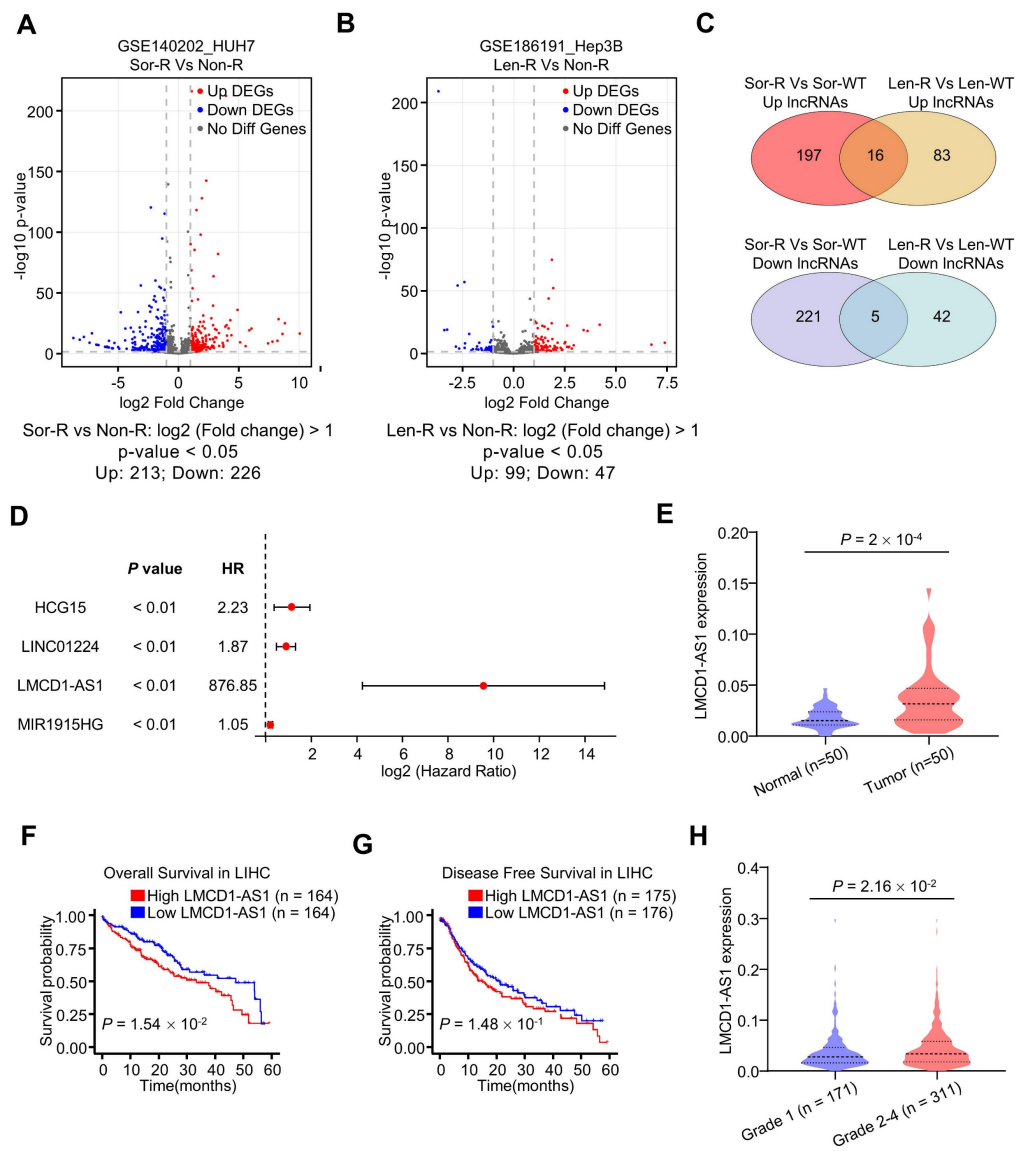
** Identification of LMCD1-AS1 as multikinase inhibitor resistance-associated lncRNA in hepatocellular carcinoma.** (A) Volcano plots showing differentially expressed lncRNAs between sorafenib-resistant (Sor-R) and non-resistant (Non-R) HUH7 cells. (B) Volcano plots showing differentially expressed lncRNAs between lenvatinib-resistant (Len-R) and non-resistant Hep3B cells. (C) Venn diagrams showing common differentially expressed lncRNAs in GEO database. (D) Forest plot showing significant positive correlation between candidate lncRNAs (including LMCD1-AS1) and overall survival (OS) in the TCGA-LIHC (liver hepatocellular carcinoma) cohort. (E) LMCD1-AS1 expression in normal and HCC tissues from the TCGA-LIHC dataset. (F and G) The correlation between LMCD1-AS1 levels and OS (F) and disease-free survival (DFS) (G) in the TCGA-LIHC cohort. (H) Relationship between LMCD1-AS1 expression and HCC tumor grade in the TCGA dataset. (**P < 0.01).

To identify sorafenib resistance-related lncRNAs, we analyzed gene expression profiles of human HCC HUH7 cells and their sorafenib-resistant subpopulation from the GSE140202 dataset (Fig. 1A). This analysis revealed 213 upregulated and 226 downregulated lncRNAs in sorafenib-resistant HUH7 cells. Similarly, analysis of the GSE186191 dataset identified 99 upregulated and 47 downregulated lncRNAs in lenvatinib-resistant Hep3B cells (Fig. 1B). Further intersection of these datasets identified sixteen commonly upregulated and five commonly downregulated lncRNAs (Fig. 1C). Univariate Cox regression analysis using the TCGA-LIHC (liver hepatocellular carcinoma) dataset demonstrated that LMCD1-AS1 was significantly associated with overall survival (OS) (Fig. 1D). Compared to normal tissues, LMCD1-AS1 expression was significantly increased in HCC tissues from the TCGA-LIHC dataset (Fig. 1E). High LMCD1-AS1 expression was significantly associated with shorter OS in TCGA-LIHC patients (Fig. 1F). Although not statistically significant, a trend toward shorter disease-free survival (DFS) was observed in patients with high LMCD1-AS1 expression (Fig. 1G). Finally, LMCD1-AS1 expression was significantly elevated in LIHC patients with advanced-stage disease compared to those with early-stage disease (Fig. 1H).

### LMCD1-AS1 attenuates MKI sensitivity and apoptosis in HCC cells

**Figure 2:**
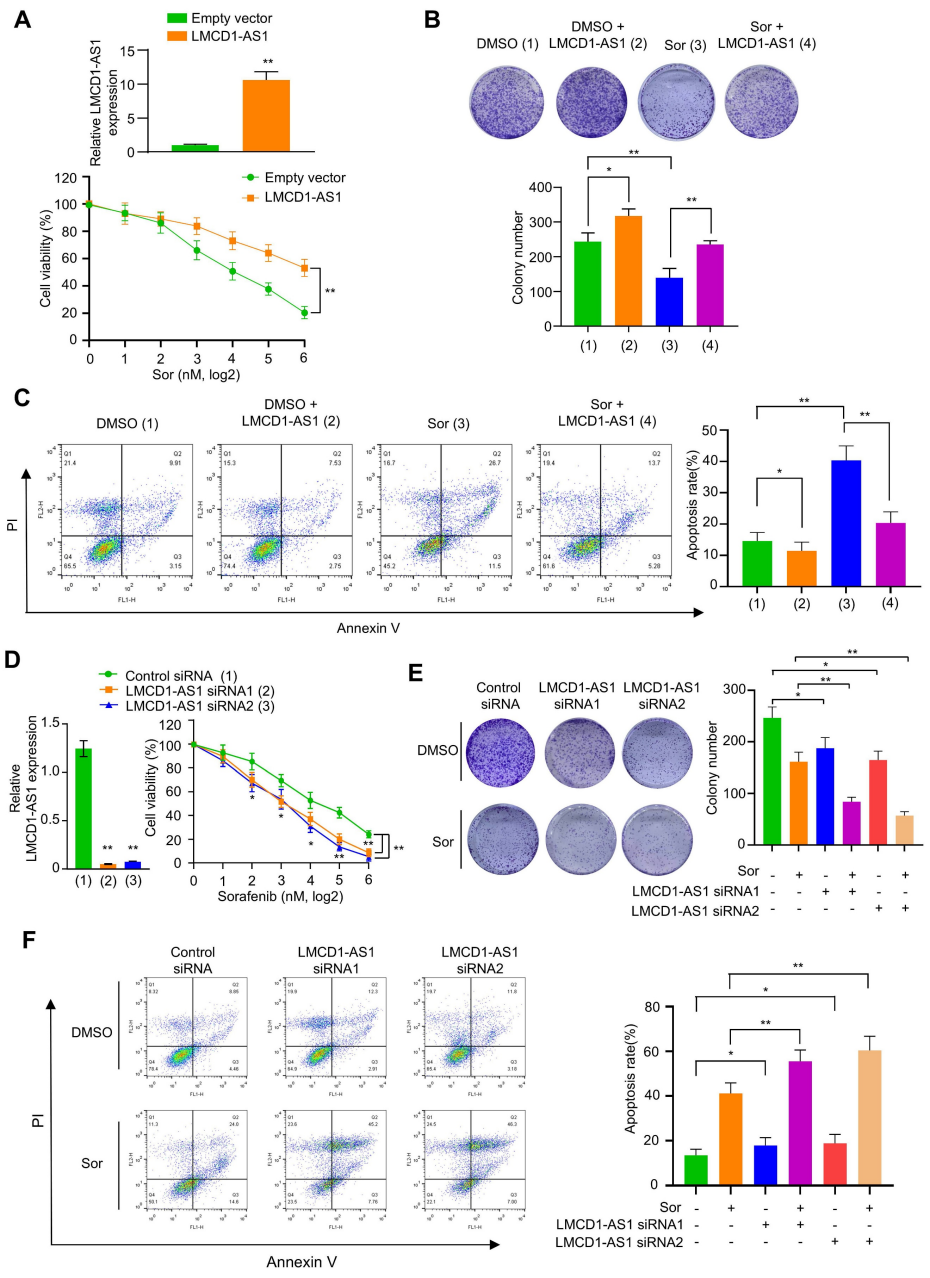
**LMCD1-AS1 confers sorafenib resistance in HepG2 cells.** (A) Cell viability of HepG2 cells stably overexpressing LMCD1-AS1 or empty vector following treatment with increasing concentrations of sorafenib. RT-qPCR shows the expression of LMCD1-AS1. (B and C) Colony formation capability (B) and apoptosis analysis by flow cytometry (C) in cells from (A). (D) Cell viability of HepG2 cells transfected with LMCD1-AS1-targeting siRNAs or control siRNA after sorafenib treatment. RT-qPCR shows the expression of LMCD1-AS1. (E and F) Colony formation (E) and apoptosis (F) in cells from (D). Data shown are mean ± SD of three independent experiments (*P < 0.05, **P < 0.01).

Next, we investigated the effect of LMCD1-AS1 on multikinase inhibitor sensitivity in HCC cells. Overexpression of LMCD1-AS1 significantly decreased sorafenib sensitivity in HepG2 and MHCC-97H cells compared to cells transfected with empty vector (Fig. 2A, B and Fig. S1A, B). HCC cells overexpressing LMCD1-AS1 showed a significantly higher IC₅₀ for sorafenib than empty-vector controls (Fig. S1C). Moreover, forced overexpression of LMCD1-AS1 markedly promoted HCC cell proliferation (Fig. S1D). Cell apoptosis assays showed that sorafenib significantly increased the apoptosis rate in these cells. While overexpression of LMCD1-AS1 alone only slightly reduced the basal apoptosis rate, it effectively attenuated sorafenib-induced apoptosis (Fig. 2C). Conversely, LMCD1-AS1 knockdown increased the sensitivity of HCC cells to sorafenib (Fig. 2D, E and Fig. S1E, F). LMCD1-AS1 knockdown significantly reduced the sorafenib IC₅₀ compared with control cells (Fig. S1G). Moreover, LMCD1-AS1 knockdown markedly inhibited HCC cell proliferation (Fig. S1H). Additionally, knockdown of LMCD1-AS1 enhanced sorafenib-induced apoptosis relative to control cells (Fig. 2F). Neither overexpression nor knockdown of LMCD1-AS1 altered LMCD1 expression. These findings indicate that LMCD1-AS1 exerts its oncogenic effects independently of LMCD1 regulation (Fig. S1I, J). Similar effects were observed in HCC cells treated with lenvatinib (Fig. S2A-F). Interestingly, both sorafenib and lenvatinib treatment elevated LMCD1-AS1 expression in HCC cells (Fig. S2G, H). Collectively, these data indicate that LMCD1-AS1 confers resistance to both sorafenib and lenvatinib in HCC cells.

### LMCD1-AS1 directly interacts with PHF8 protein

**Figure 3:**
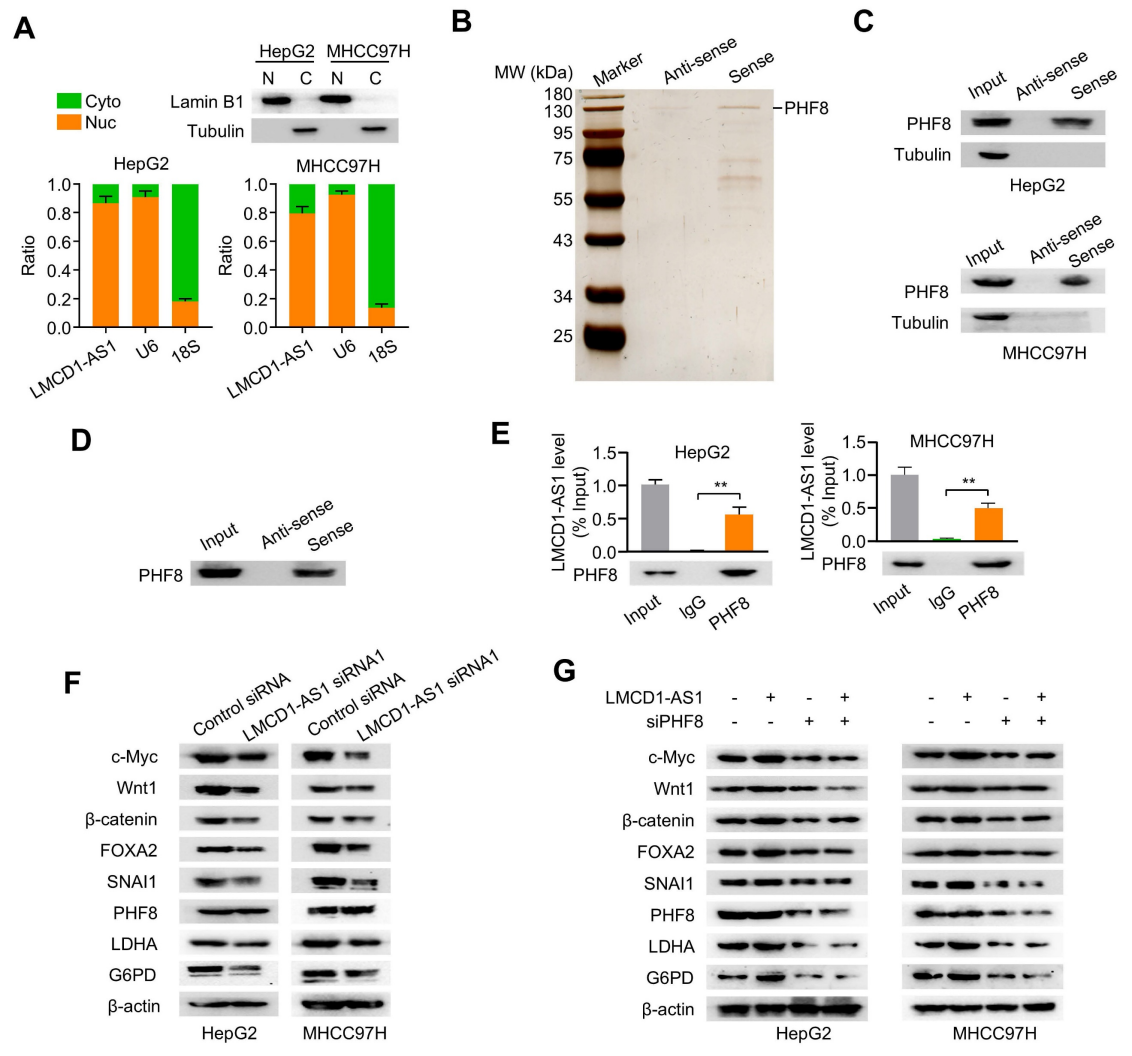
** LMCD1-AS1 physically and functionally interacts with the epigenetic regulator PHF8.** (A) RT-qPCR analysis of the ratio of nuclear and cytoplasmic LMCD1-AS1 expression in HepG2 and MHCC-97H cells. Lamin B1 and α-tubulin served as nuclear and cytoplasmic protein markers, respectively; U6 snRNA and 18S rRNA served as nuclear and cytoplasmic RNA markers, respectively. Cyto (C), cytoplasm. Nuc (N), nucleus. (B) Identification of LMCD1-AS1-interacting proteins by RNA pull-down assay coupled with SDS-PAGE and silver staining. (C) Validation of LMCD1-AS1-PHF8 interaction by RNA pull-down followed by immunoblotting for PHF8 in HepG2 and MHCC-97H cells using biotinylated sense and antisense LMCD1-AS1 transcripts. (D) Direct binding between LMCD1-AS1 and recombinant PHF8 protein confirmed by RNA pull-down and immunoblotting. (E) RIP assays using anti-PHF8 antibody or normal IgG control in HepG2 and MHCC-97H cells. (F) Western blot analysis of indicated proteins in HepG2 and MHCC-97H cells transfected with LMCD1-AS1 siRNA1 or control siRNA. (G) Western blot analysis of indicated proteins in HepG2 and MHCC-97H cells transfected with LMCD1-AS1, PHF8 siRNA (siPHF8) or LMCD1-AS1 plus siPHF8. Empty vector for LMCD1-AS1 and control siRNA were used as a control. Data shown are mean ± SD of three independent experiments (*P < 0.05, **P < 0.01).

Subcellular fractionation assays showed that LMCD1-AS1 was localized predominantly in the nucleus of HepG2 and MHCC-97H cells (Fig. 3A). To identify LMCD1-AS1-interacting proteins, we performed RNA pull-down assays coupled with mass spectrometry (MS). MS analysis identified PHF8 as the most significantly enriched binding partner based on its highest protein score (Fig. 3B and Table S3). Given that PHF8 is a histone lysine demethylase frequently overexpressed in cancers 23, we selected it for further study. RNA pull-down assays confirmed specific interaction between LMCD1-AS1 and PHF8, but not α-tubulin, in both cell lines (Fig. 3C). Direct binding was further demonstrated using recombinant PHF8 protein (Fig. 3D). Moreover, RNA immunoprecipitation (RIP) assays showed marked enrichment of LMCD1-AS1 in PHF8 immunoprecipitates (Fig. 3E). Collectively, these results demonstrate a direct physical interaction between LMCD1-AS1 and PHF8.

### LMCD1-AS1 promotes PHF8 target gene expression in a PHF8-dependent manner

Given the physical interaction between LMCD1-AS1 and PHF8, we investigated whether LMCD1-AS1 modulates expression of PHF8-regulated genes. Western blot analysis revealed that LMCD1-AS1 knockdown significantly reduced protein levels of PHF8 target genes (c-Myc, Wnt1, β-catenin, FOXA2, SNAI1, LDHA, and G6PD) without affecting PHF8 expression in HCC cells (Fig. 3F). To determine if this regulation is mediated through PHF8, we overexpressed LMCD1-AS1 and observed concomitant upregulation of these target genes (Fig. 3G). Critically, PHF8 silencing with previously validated PHF8 siRNA completely abrogated the ability of LMCD1-AS1 overexpression to induce target gene expression (Fig. 3G), demonstrating that LMCD1-AS1 enhances PHF8 target gene expression via PHF8.

### LMCD1-AS1 facilitates PHF8-histone H4 complex formation to reduce H4K20me1 levels

**Figure 4:**
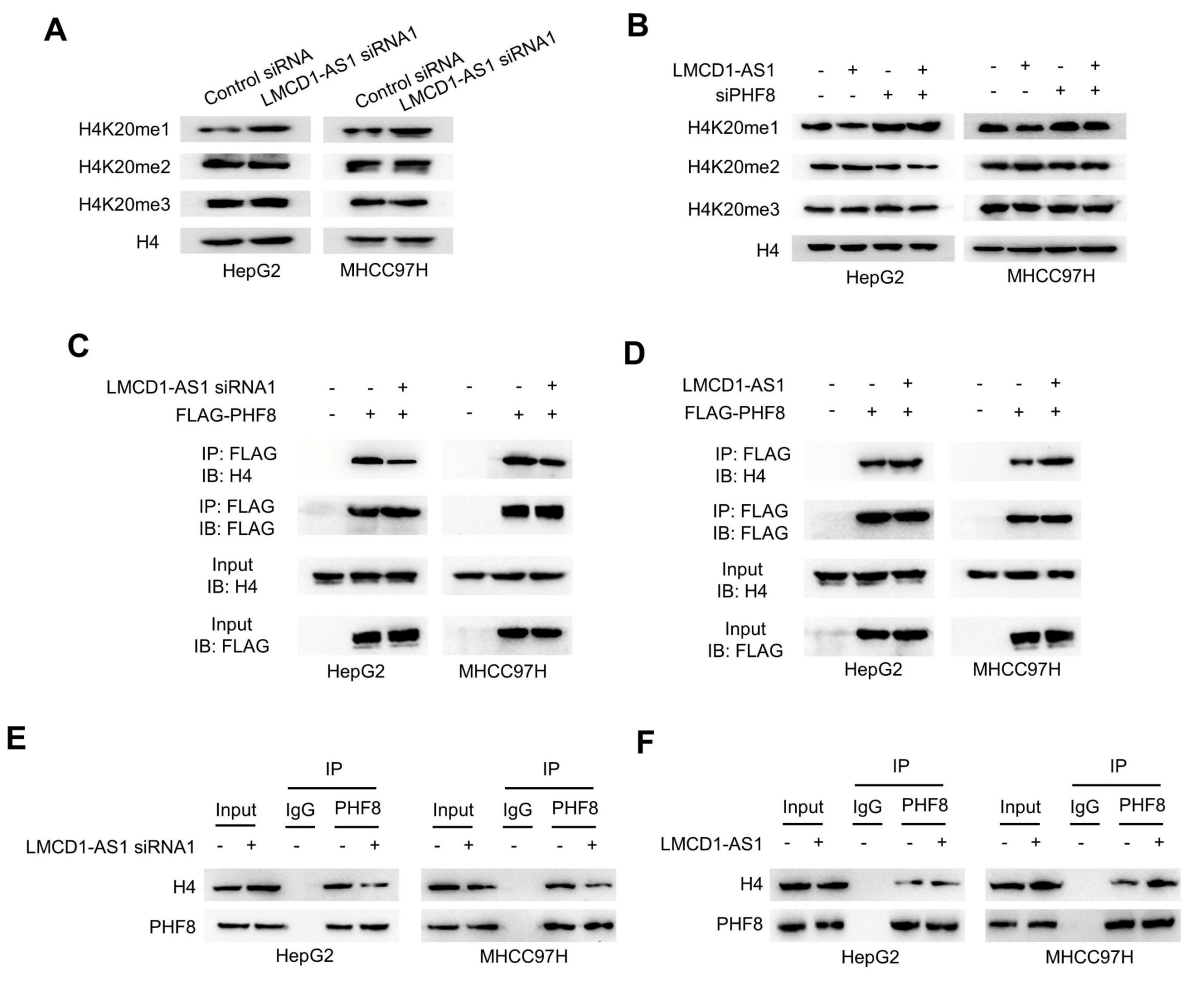
** LMCD1-AS1 facilitates PHF8-histone H4 complex formation and reduces H4K20me1 levels.** (A) Western blot analysis of H4K20me1 levels in HepG2 and MHCC97H cells transfected with LMCD1-AS1 siRNA1 or control siRNA. (B) Western blot analysis of H4K20me1 levels in HepG2 and MHCC97H cells transfected with LMCD1-AS1, siPHF8 or LMCD1-AS1 plus siPHF8. Empty vector and control siRNA were used as a control. (C) Co-IP analysis of PHF8-H4 interaction in HepG2 and MHCC97H cells transfected with LMCD1-AS1 siRNA1 and FLAG-tagged PHF8. IP, immunoprecipitation. IB, immunoblot. (D) Co-IP analysis of PHF8-H4 interaction in HepG2 and MHCC97H cells transfected with LMCD1-AS1 and FLAG-tagged PHF8. (E and F) Co-IP analysis of HepG2 and MHCC-97H cells transfected with LMCD1-AS1 siRNA1 (E) or LMCD1-AS1 (F) and immunoprecipitated with anti-PHF8 or normal IgG.

As PHF8 regulates H4K20 demethylation in cell fate determination, we examined whether LMCD1-AS1 modulates H4K20 methylation. LMCD1-AS1 knockdown significantly increased H4K20me1 levels in HepG2 and MHCC-97H cells compared with controls, without affecting H4K20me2 or H4K20me3 levels (Fig. 4A). Conversely, LMCD1-AS1 overexpression reduced H4K20me1 levels, whereas PHF8 silencing elevated H4K20me1 without altering H4K20me2 or H4K20me3 levels, and abolished LMCD1-AS1-mediated reduction of H4K20me1 (Fig. 4B).

To elucidate the mechanism underlying LMCD1-AS1-dependent H4K20me1 reduction, we assessed its effect on interaction between PHF8 and histone H4 in HepG2 and MHCC-97H cells. Co-IP showed that LMCD1-AS1 knockdown impaired exogenous PHF8-H4 complex formation (Fig. 4C), whereas LMCD1-AS1 overexpression enhanced this interaction (Fig. 4D). Moreover, Co-IP confirmed endogenous PHF8-H4 binding (Fig. 4E, F). Collectively, these data demonstrate that LMCD1-AS1 promotes PHF8-histone H4 complex assembly to facilitate H4K20me1 demethylation.

### LMCD1-AS1 promotes lactate production and increases the NAD⁺/NADH ratio in HCC cells

**Figure 5:**
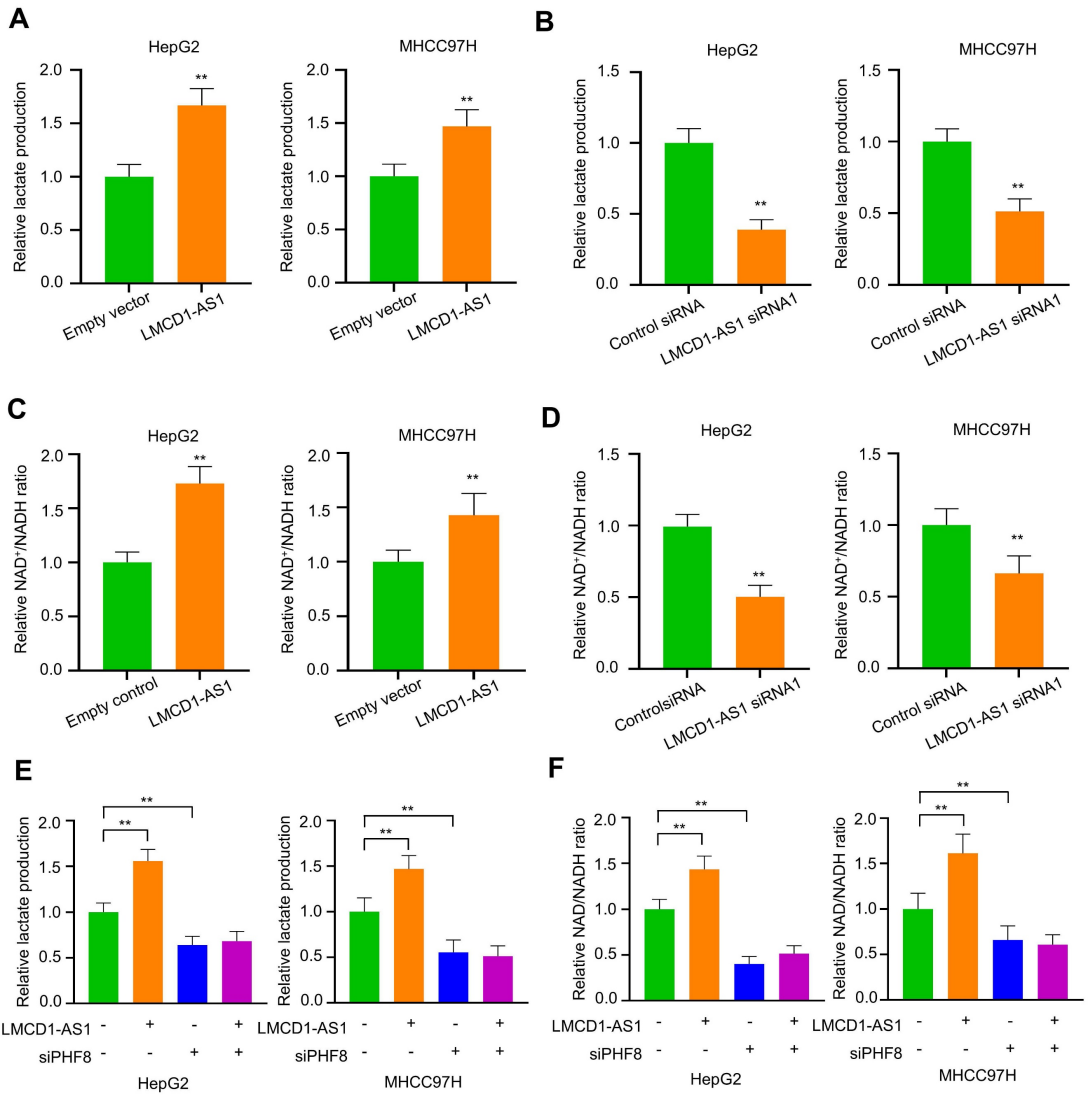
** LMCD1-AS1 promotes lactate production and increases the NAD⁺/NADH ratio via PHF8 in HCC cells.** (A and B) Measurement of lactate production in HepG2 and MHCC97H cells transfected with LMCD1-AS1 (A) or LMCD1-AS1 siRNA1 (B). (C and D) NAD⁺/NADH ratio in HepG2 and MHCC97H cells following transfection with LMCD1-AS1 (C) or LMCD1-AS1 siRNA1 (D). (E and F) Measurement of lactate production (E) and NAD⁺/NADH ratio (F) in HepG2 and MHCC97H cells transfected with LMCD1-AS1, siPHF8 or LMCD1-AS1 plus siPHF8. Data shown are mean ± SD of three independent experiments (*P < 0.05, **P < 0.01).

Elevated lactate within the tumor microenvironment is a key driver of resistance to sorafenib and lenvatinib 29. Targeting lactate production—specifically by inhibiting LDHA, which catalyzes pyruvate-to-lactate conversion coupled with NADH oxidation to NAD⁺ during aerobic glycolysis—represents a promising strategy to overcome or prevent resistance to these tyrosine kinase inhibitors in cancer. Given that LMCD1-AS1 promotes LDHA expression, we investigated whether LMCD1-AS1 regulates lactate production and the NAD⁺/NADH ratio in HCC cells. As expected, overexpression of LMCD1-AS1 increased lactate production (Fig. 5A), while knockdown of LMCD1-AS1 reduced lactate production (Fig. 5B). Similar effects on the NAD⁺/NADH ratio were observed (Fig. 5C, D).

As LMCD1-AS1 enhances LDHA expression via PHF8, we investigated whether PHF8 regulates lactate production and the NAD⁺/NADH ratio in HCC cells. Interestingly, PHF8 silencing reduced lactate production and abolished LMCD1-AS1-mediated lactate production (Fig. 5E). Similar effects on the NAD⁺/NADH ratio were observed (Fig. 5F). These data suggest that lactate metabolism may contribute to LMCD1-AS1-mediated sorafenib and lenvatinib resistance in HCC cells.

### PHF8 silencing reverses LMCD1-AS1-enhanced MKI resistance in HCC cells

**Figure 6:**
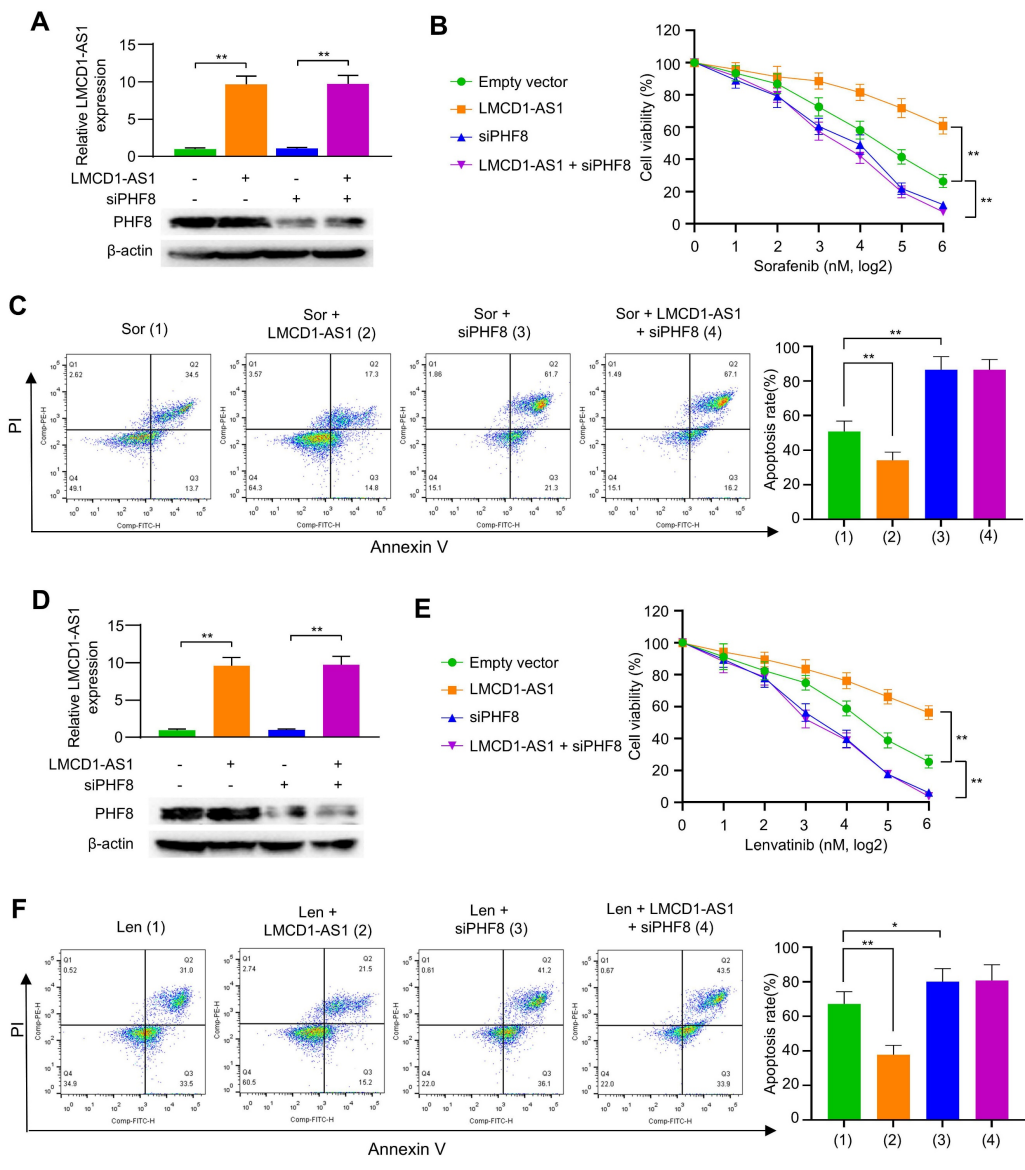
** LMCD1-AS1 induces resistance to sorafenib and lenvatinib through PHF8.** (A) RT-qPCR and Western blot analysis of LMCD1-AS1 and PHF8 expression in HepG2 cells transfected with LMCD1-AS1, siPHF8 or LMCD1-AS1 plus siPHF8. (B and C) Cell viability (B) and apoptosis analysis by flow cytometry (C) in HepG2 cells transfected as in (A) and treated with sorafenib. (D) RT-qPCR and Western blot analysis of LMCD1-AS1 and PHF8 expression in MHCC97H cells transfected with LMCD1-AS1, siPHF8 or LMCD1-AS1 plus siPHF8. (E and F) Cell viability (E) and apoptosis analysis by flow cytometry (F) in MHCC97H cells transfected as in (D) and treated with lenvatinib. Data shown are mean ± SD of three independent experiments (*P < 0.05, **P < 0.01).

Given the role of LMCD1-AS1 in promoting MKI resistance, we investigated whether this occurs through PHF8. LMCD1-AS1 overexpression conferred sorafenib resistance in HCC cells, whereas PHF8 silencing not only reduced basal resistance but also completely abolished LMCD1-AS1-driven resistance (Fig. 6A, B and Fig. S3A). Consistently, LMCD1-AS1 overexpression attenuated sorafenib-induced apoptosis in HCC cells, an effect counteracted by PHF8 silencing (Fig. 6C). Identical rescue effects were observed with lenvatinib treatment (Fig. 6D-F and Fig. S3B). These data establish PHF8 as the essential mediator of LMCD1-AS1-induced therapeutic resistance to sorafenib and lenvatinib in HCC.

### LMCD1-AS1 overexpression reduces sorafenib sensitivity in HCC xenografts

**Figure 7:**
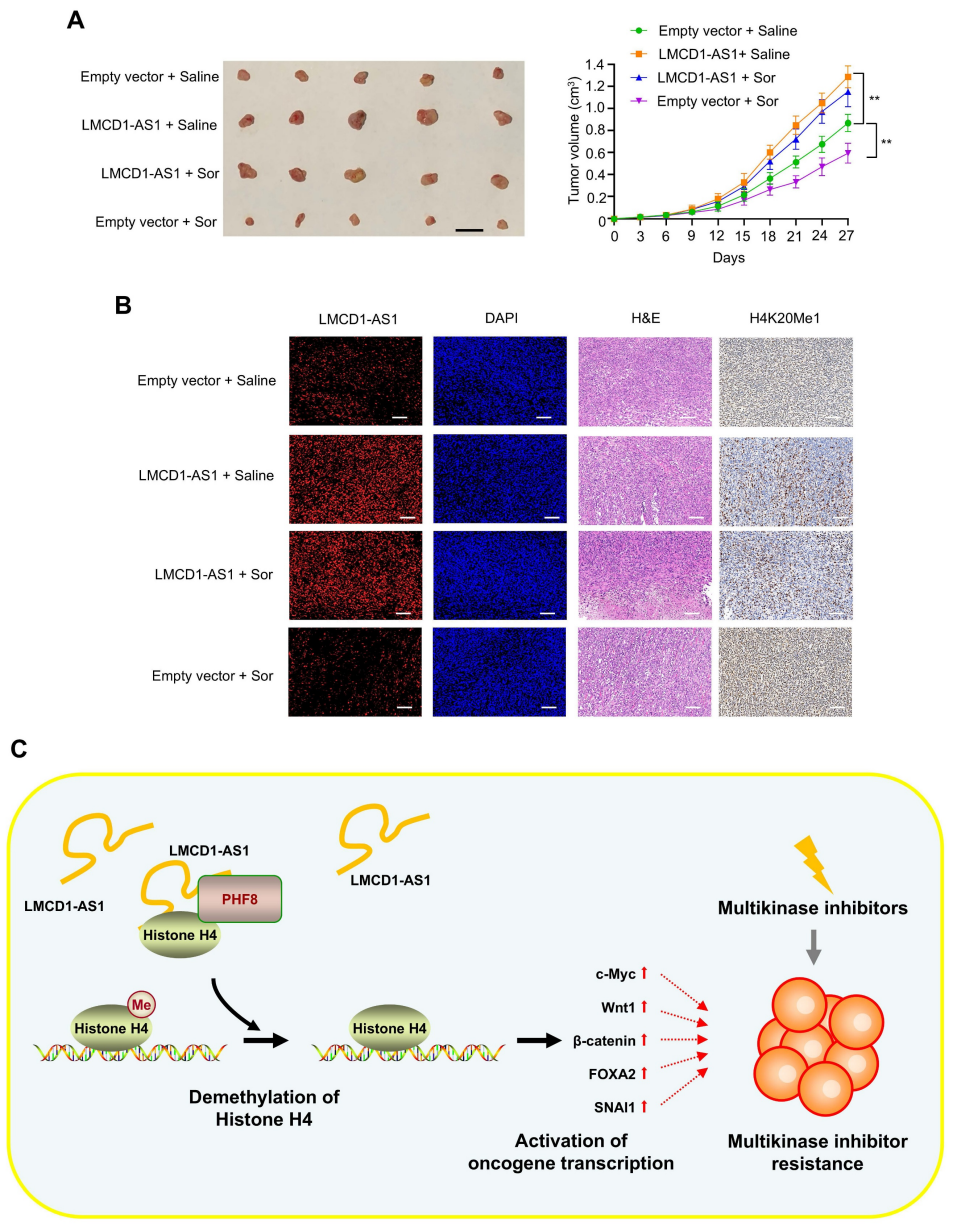
** LMCD1-AS1 promotes sorafenib-resistant tumor growth in a HepG2 xenograft mouse model.** (A) NTG mice were subcutaneously injected with HepG2 cells stably expressing LMCD1-AS1 or empty vector into the right flank, followed by intraperitoneal administration of sorafenib. Tumor volume was measured at the indicated time points using a vernier caliper. (B) Representative fluorescence in situ hybridization (FISH) images showing LMCD1-AS1 expression and immunohistochemical (IHC) staining of H4K20me1 in representative HepG2 tumor tissues. Scale bar: 100 μm. (C) A proposed model illustrating that LMCD1-AS1 facilitates PHF8-histone H4 complex formation and promotes H4K20me1 demethylation, leading to multikinase inhibitor resistance in hepatocellular carcinoma. Tumor volumes were presented as means ± SD (n = 5). Scale bar for tumors, 10 mm. (*P < 0.05, **P < 0.01).

Next, to investigate whether LMCD1-AS1 regulates sorafenib sensitivity *in vivo*, we subcutaneously injected HepG2 cells stably expressing either an empty vector control or LMCD1-AS1 into 6-week-old male nude mice. Once tumors became palpable, mice in each group were administered either sorafenib or normal saline via intraperitoneal injection once weekly for three weeks. Sorafenib treatment significantly inhibited tumor growth compared to the saline control group, while LMCD1-AS1 overexpression itself promoted tumor growth (Fig. 7A). Notably, LMCD1-AS1 overexpression attenuated the inhibitory effect of sorafenib on tumor growth. Consistent with our *in vitro* findings, HepG2 tumors overexpressing LMCD1-AS1 exhibited decreased H4K20me1 levels (Fig. 7B).

## Discussion

Hepatocellular carcinoma patients treated with first-line MKIs such as sorafenib and lenvatinib invariably develop therapeutic resistance. Solving the problem of MKI resistance is a big challenge for the treatment of HCC. Our study uncovers the lncRNA LMCD1-AS1 as a critical regulator of MKI resistance through an integrated epigenetic-metabolic axis (Fig. 7C). Clinically, elevated LMCD1-AS1 expression correlates with advanced tumor stage and poor survival in HCC patients, positioning it as a promising prognostic biomarker. Functionally, we demonstrate that LMCD1-AS1 overexpression blunts sorafenib- and lenvatinib-induced apoptosis, while its genetic ablation restores drug sensitivity—establishing its role as a shared resistance factor for these therapeutically distinct MKIs.

Mechanistically, LMCD1-AS1 operates by directly binding the histone demethylase PHF8, a partnership validated through RNA pull-down, RIP assays, and recombinant protein interaction studies. This physical interaction facilitates PHF8-histone H4 complex assembly, enhancing H4K20me1 demethylation—an epigenetic alteration previously unreported in MKI resistance contexts. Subsequent erosion of H4K20me1 marks derepresses oncogenic transcriptional programs, including c-Myc, Wnt/β-catenin, and notably LDHA, a key glycolytic enzyme. Crucially, LMCD1-AS1-driven LDHA upregulation fuels lactate overproduction and alters the NAD^+^/NADH ratio, creating an acidified tumor microenvironment conducive to drug resistance 30-32. The centrality of PHF8 in this cascade is underscored by our finding that PHF8 silencing completely abrogates LMCD1-AS1-mediated resistance, abolishing both its epigenetic and metabolic effects.

Our work connects two emerging paradigms in therapy resistance. First, while lncRNA BBOX1-AS1 is known to stabilize PHF8 mRNA by targeting its inhibitor miR-361-3p in HCC 33, LMCD1-AS1 represents a novel scaffold that enhances PHF8's catalytic activity towards H4K20me1—a mark linked to chromatin compaction and transcriptional silencing 34,35. Second, by linking H4K20me1 demethylation to lactate metabolism via LDHA, we reveal how epigenetic reprogramming directly potentiates the Warburg effect, wherein cancer cells preferentially rely on glycolysis for ATP production (even under aerobic conditions) to meet heightened metabolic demands 36,37. This "epi-metabolic" crosstalk distinguishes LMCD1-AS1 from resistance-associated lncRNAs like LINC00152 and others operating through isolated pathways 38,39. Critically, *in vivo* validation confirms that LMCD1-AS1 overexpression accelerates tumor growth and impairs sorafenib efficacy, mirroring clinical observations of its association with advanced disease.

The translational potential of this PHF8 axis is significant, particularly as PHF8 overexpression is highly prevalent in cancers, including HCC. Therefore, it is important to inhibit PHF8 activity to inhibit tumor growth and enhance MKI sensitivity. Since LMCD1-AS1 confers PHF8-mediated MKI resistance and promotes LDHA expression, specific PHF8 inhibitors or LDHA antagonists may represent rational candidates to counteract LMCD1-AS1-driven resistance 40,41. Moreover, detecting LMCD1-AS1 in liquid biopsies could identify patients prone to MKI failure prior to treatment initiation. Future studies should address whether H4K20me1 reduction expands beyond LDHA to modulate other resistance determinants (e.g., drug efflux pumps) and how LMCD1-AS1-generated lactate influences immune cell function in the tumor microenvironment. Prospective clinical cohorts validating LMCD1-AS1's predictive value are also warranted.

In summary, we identify LMCD1-AS1 as a master regulator of MKI resistance in HCC, coordinating PHF8-dependent H4K20me1 demethylation and LDHA-mediated metabolic rewiring. Disrupting this newly defined "epi-metabolic" axis offers a compelling strategy to extend the efficacy of first-line HCC therapies.

## Supplementary Material

Supplementary figures and tables.
