# Supplementary material for: LncRNA LMCD1-AS1 Interacts with PHF8 to Promote Hepatocellular Carcinoma Resistance to Multikinase Inhibitors
Source: Int J Biol Sci. 2026 Mar 30;22(8):3990–4003. doi: 10.7150/ijbs.129651 (PMC13137860; doi:10.7150/ijbs.129651)
Supplement: Supplementary file 1 — Supplementary figures and tables. [file ijbsv22p3990s1.pdf]

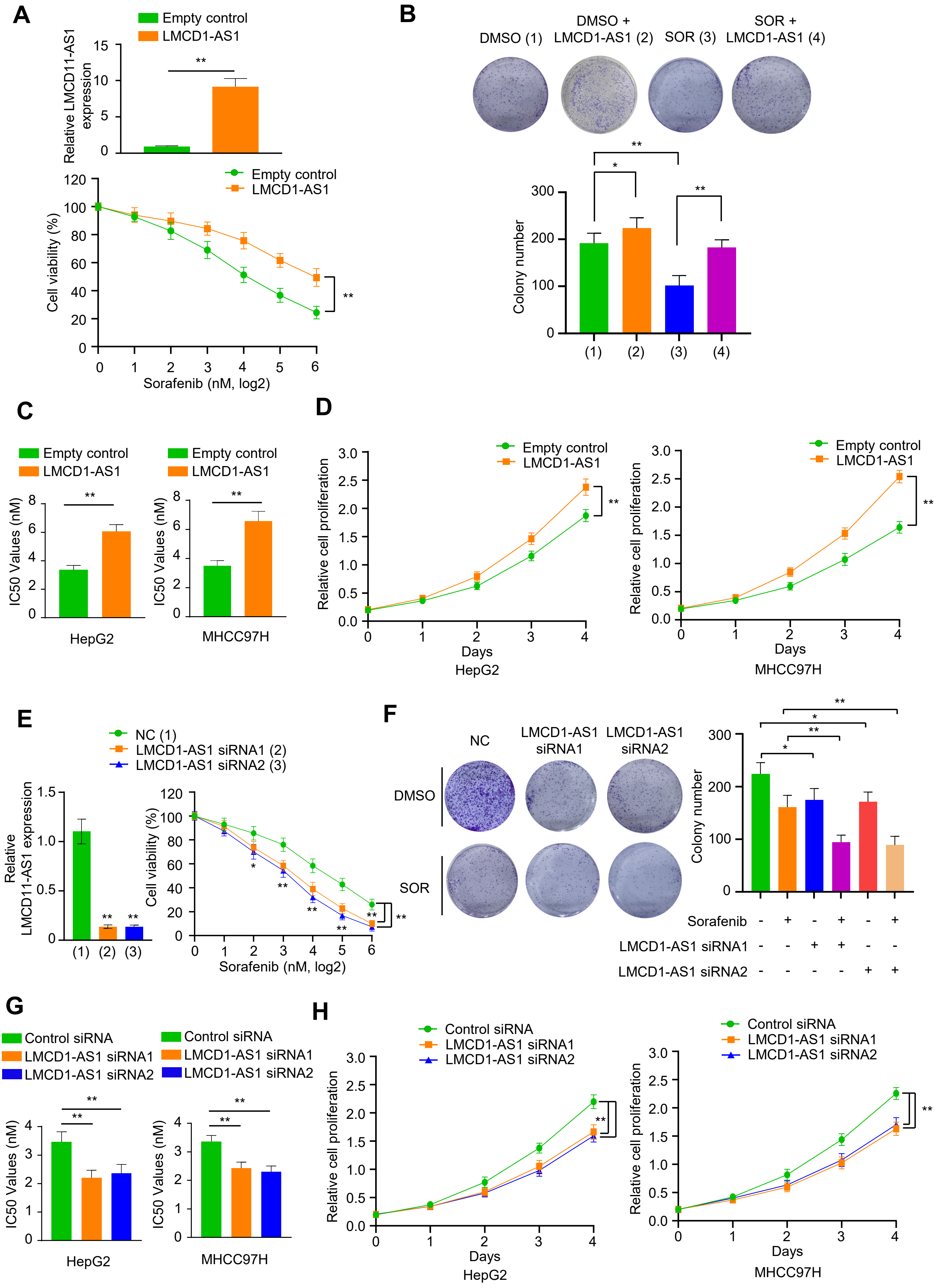
A
Empty control
LMCD1-AS1
Relative LMCD1-AS1 expression
Cell viability (%)
Sorafenib (nM, log2)
B
DMSO (1)
DMSO + LMCD1-AS1 (2)
SOR (3)
SOR + LMCD1-AS1 (4)
Colony number
C
IC50 Values (nM)
HepG2
MHCC97H
D
Relative cell proliferation
Days
E
NC (1)
LMCD1-AS1 siRNA1 (2)
LMCD1-AS1 siRNA2 (3)
Relative LMCD11-AS1 expression
F
NC
LMCD1-AS1 siRNA1
LMCD1-AS1 siRNA2
DMSO
SOR
Sorafenib
G
Control siRNA
H

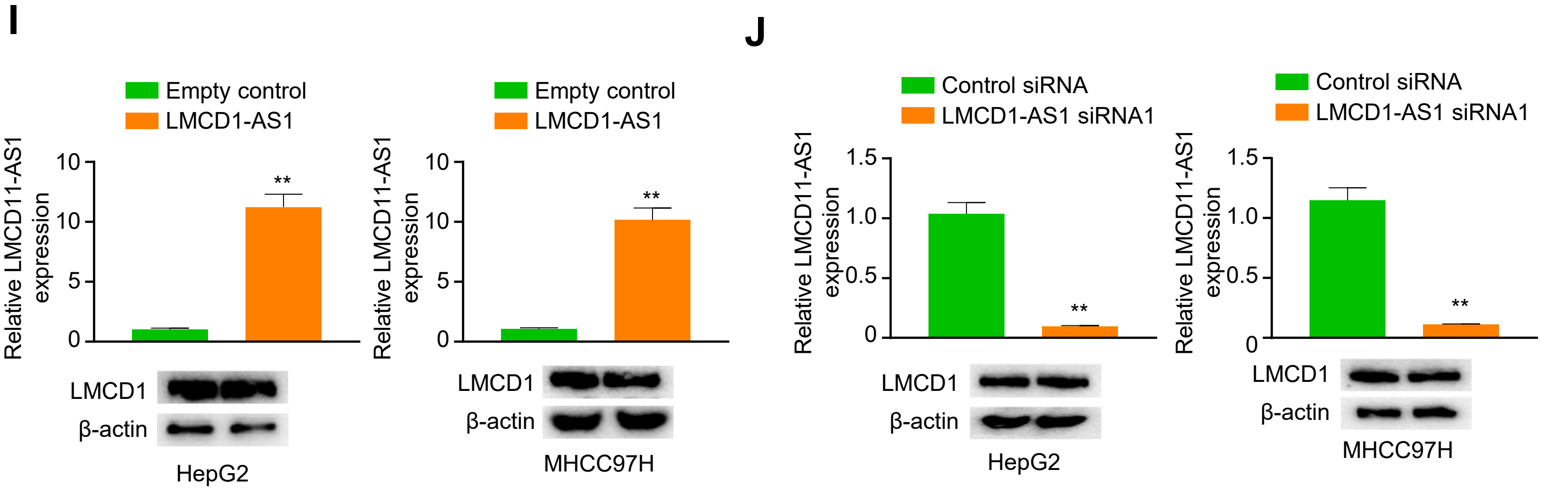

Fig S1. LMCD1-AS1 confers sorafenib resistance in MHCC-97H cells. (A) Cell viability of MHCC-97H cells stably expressing LMCD1-AS1 or empty vector following treatment with increasing concentrations of sorafenib. RT-qPCR shows the expression of LMCD1-AS1. (B) Colony formation capability in cells from (A). (C) IC50 Values of the indicated HCC cells stably expressing LMCD1-AS1 or empty vector. (D) Cell proliferation of the indicated HCC cells stably expressing LMCD1-AS1 or empty vector. (E) Cell viability of MHCC-97H cells transfected with LMCD1-AS1-targeting siRNAs or control siRNA after sorafenib treatment. RT-qPCR shows the expression of LMCD1-AS1. (F) Colony formation capability in cells from (E). (G) IC50 Values of the indicated HCC cells transfected with LMCD1-AS1-targeting siRNAs or control siRNA. (H) Cell proliferation of the indicated HCC cells transfected with LMCD1-AS1-targeting siRNAs or control siRNA. (I) Western blot analysis of LMCD1 levels in the indicated HCC cells transfected with LMCD1-AS1 or empty vector. RT-qPCR shows the expression of LMCD1-AS1. (J) Western blot analysis of LMCD1 levels in the indicated HCC cells transfected with LMCD1-AS1 siRNA1 or control siRNA. RT-qPCR shows the expression of LMCD1-AS1. Data shown are mean ± SD of three independent experiments (*P < 0.05, **P < 0.01).

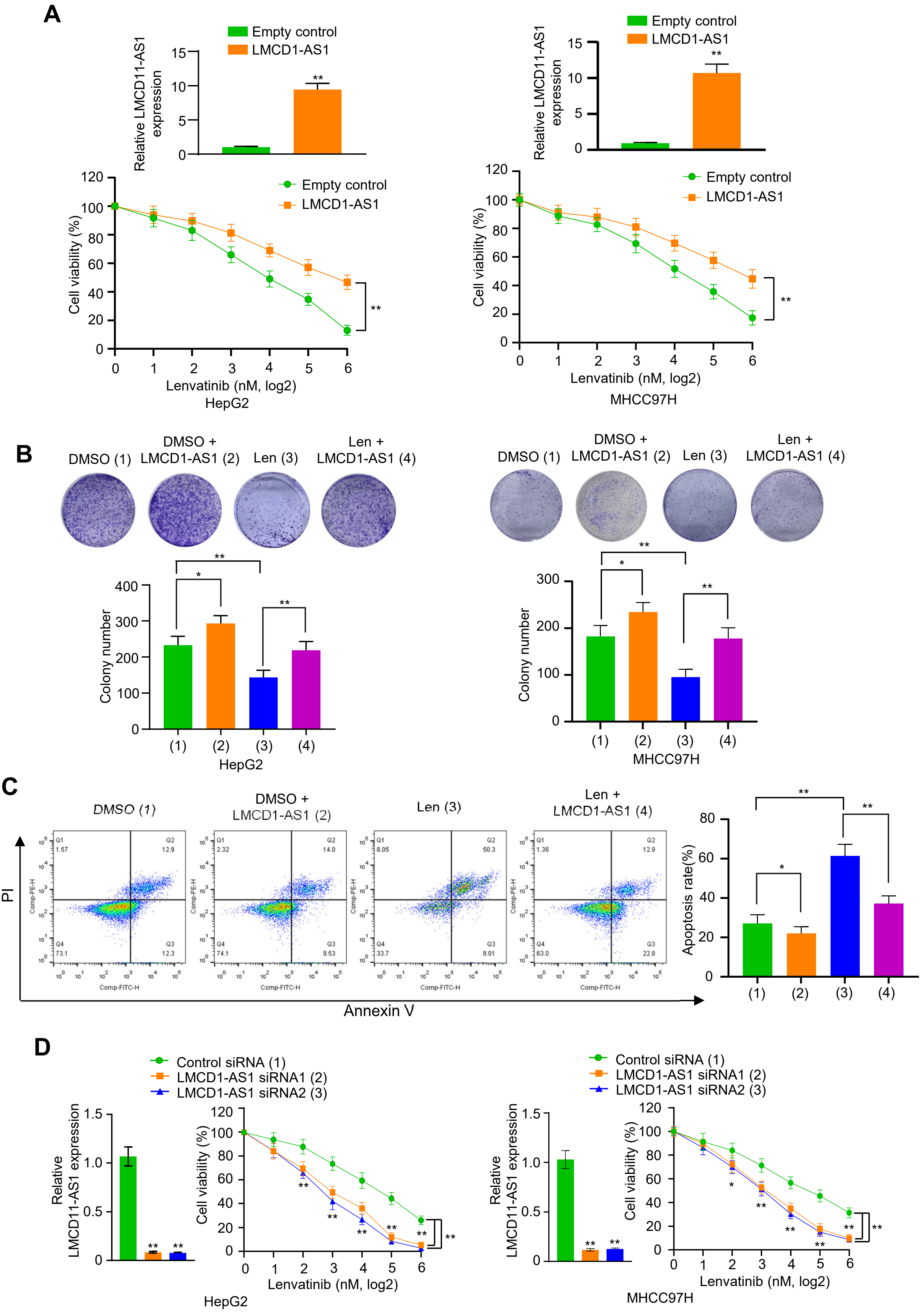

A
Empty control
LMCD1-AS1
Relative LMCD11-AS1 expression
Cell viability (%)
Lenvatinib (nM, log2)
HepG2
MHCC97H
B
DMSO (1)
DMSO + LMCD1-AS1 (2)
Len (3)
Len + LMCD1-AS1 (4)
Colony number
(1)
(2)
(3)
(4)
HepG2
MHCC97H
C
DMSO (1)
DMSO + LMCD1-AS1 (2)
Len (3)
Len + LMCD1-AS1 (4)
PI
Annexin V
Comp-PE-H
Comp-FITC-H
Q1 1.57
Q2 12.9
Q4 73.1
Q3 12.3
Q1 2.32
Q2 14.0
Q4 74.1
Q3 9.53
Q1 8.05
Q2 50.3
Q4 33.7
Q3 8.01
Q1 1.36
Q2 12.9
Q4 63.0
Q3 22.8
Apoptosis rate(%)
D
Control siRNA (1)
LMCD1-AS1 siRNA1 (2)
LMCD1-AS1 siRNA2 (3)
Relative LMCD11-AS1 expression
Cell viability (%)
Lenvatinib (nM, log2)
HepG2
MHCC97H

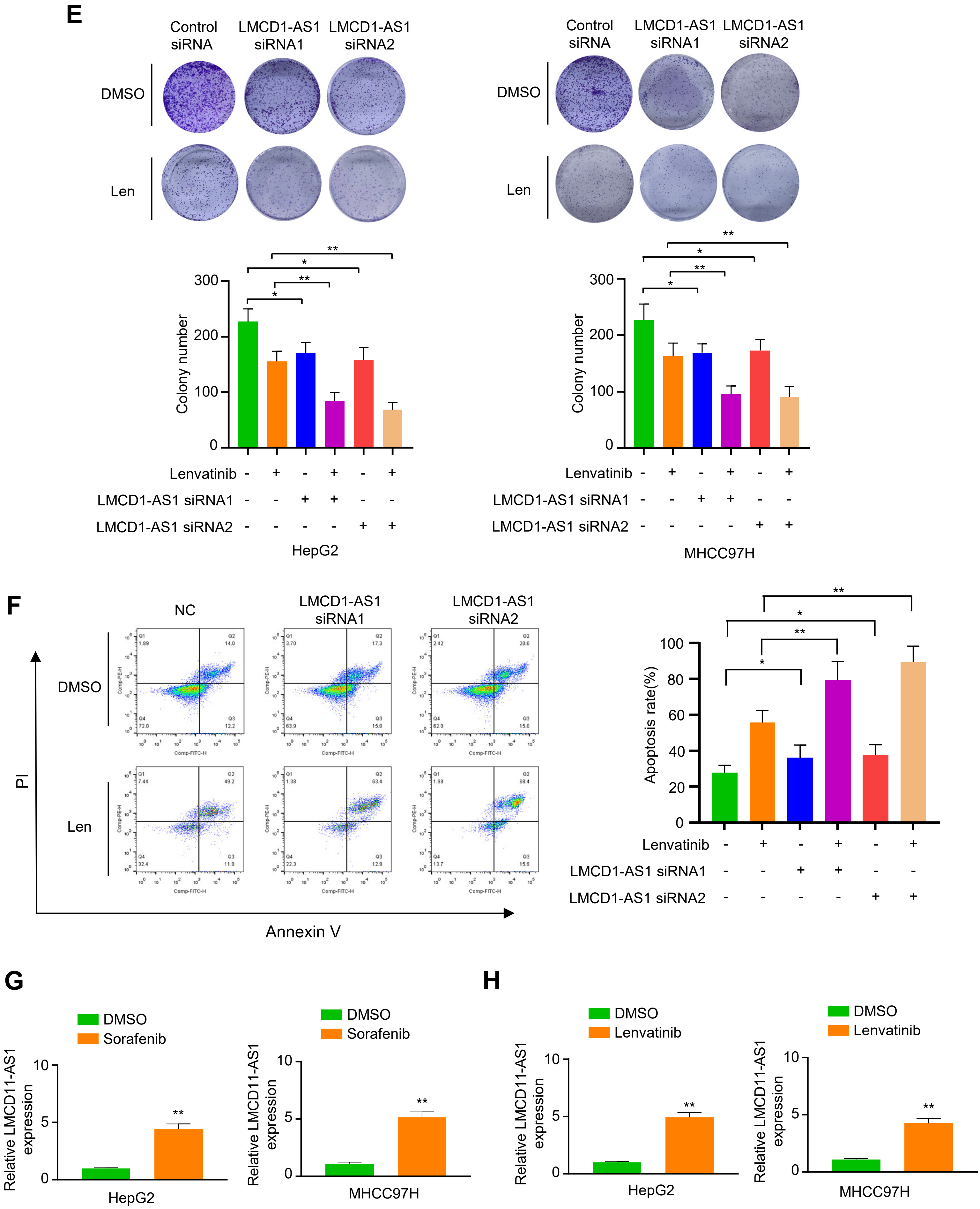

Figure S2. LMCD1-AS1 confers lenvatinib resistance in HCC cells. (A) Cell viability of HepG2 and MHCC-97H cells stably expressing LMCD1-AS1 or empty vector and treated with increasing concentrations of lenvatinib. RT-qPCR shows the expression of LMCD1-AS1. (B and C) Colony formation capability (B) and apoptosis analysis by flow cytometry (C) in cells from (A). (D) Cell viability of HepG2 and MHCC-97H cells transfected with LMCD1-AS1 siRNAs or control siRNA and treated with increasing concentrations of lenvatinib. RT-qPCR shows the expression of LMCD1-AS1. (E and F) Colony formation capability (E) and apoptosis analysis by flow cytometry (F) in cells from (D). (G and H) RT-qPCR analysis of LMCD1-AS1 expression in the indicated HCC cells treated with sorafenib (G) or Lenvatinib (H). DMSO were used as a control. Data shown are mean ± SD of three independent experiments (*$P < 0.05$, **$P < 0.01$).

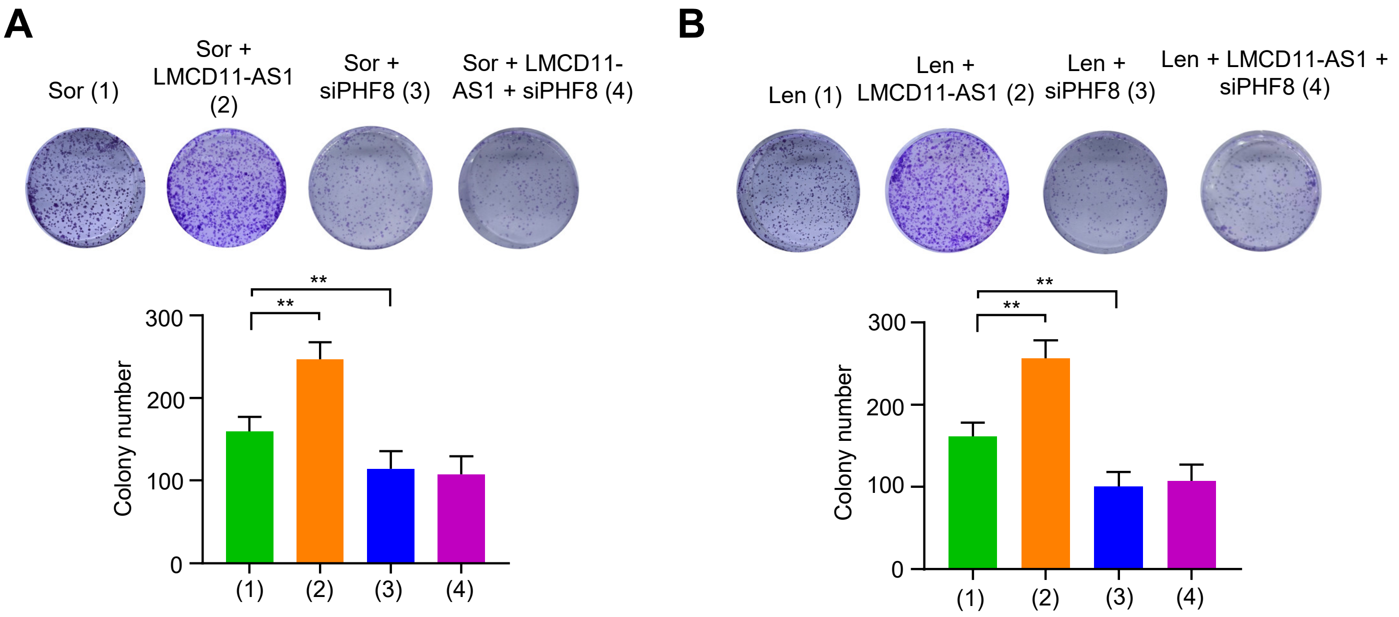

Figure S3. LMCD1-AS1 enhances the colony-forming ability of HCC cells treated with sorafenib or Lenvatinib via PHF8. (A) Colony formation assays in HepG2 cells as in Fig. 6B. (B) Colony formation assays in MHCC-97H cells as in Fig. 6E. Data shown are mean ± SD of three independent experiments (*P < 0.05, **P < 0.01).

Table S1. The cDNA target sequences of siRNAs

Gene	Sequence (5'→3')
LMCD1-AS1 siRNA-1	CAGTTTAGTTTATATATAA
LMCD1-AS1 siRNA-2	CCTATTCTGTAGAAGTAAA
PHF8 siRNA	GGAGGACTATACAACAGAT

Table S2. Primers used for RT-qPCR

	Forward(5'→3')	Reverse(5'→3')
LMCD1-AS1	AGAGCCGAGGGGACCTATATT	CCCGTGTCAAAAAGCGATGA
β-actin	ATCACCATTGGCAATGAGCG	TTGAAGGTAGTTTCGTGGAT

Table S3. Proteins identified by mass spectrometry of RNA pull down

Accession	Gene Name	Protein score	Protein description
Q9NY65	TBA8	6.49	Tubulin alpha-8 chain
Q8N8D1	PDCD7	7.97	Programmed cell death protein 7
Q9HCE1	MOV10	9	Helicase MOV-10
Q9NX24	NHP2	8.72	H/ACA ribonucleoprotein complex subunit 2
Q14247	SRC8	5.24	Src substrate cortactin
Q8N5A5	ZGPAT	5.3	Zinc finger CCCH-type with G patch domain-containing protein
P51398	RT29	9.02	Small ribosomal subunit protein mS29
Q9UPP1	PHF8	9.92	Histone lysine demethylase PHF8
P47755	CAZA2	5.57	F-actin-capping protein subunit alpha-2
Q9Y580	RBM7	7.56	RNA-binding protein 7
Q9Y2R4	DDX52	7.69	Probable ATP-dependent RNA helicase DDX52
O75817	POP7	9.09	Ribonuclease P protein subunit p20
O43324	MCA3	8.54	Eukaryotic translation elongation factor 1 epsilon-1
Q13162	PRDX4	5.86	Peroxiredoxin-4
Q9UEY8	ADDG	5.92	Gamma-adducin
Q9UBS4	DJB11	5.81	DnaJ homolog subfamily B member 11
P04004	VTNC	5.55	Vitronectin
